# Role of micronutrients, phytochemicals, and lipid metabolism in mammary gland development, lactation efficiency, and breast cancer risk

**DOI:** 10.3389/fnut.2026.1811082

**Published:** 2026-05-05

**Authors:** Jia Qi, Xiaoyuan Liang, Xiangzhi Li, Jing Zhu, Xueping Li, Yaoyi Kong, Kanghua Huang, Jun Lai, Kunpeng Hu, Xulong Fan

**Affiliations:** 1 Department of Breast and Thyroid Surgery, The Third Affiliated Hospital of Sun Yat-Sen University, Guangzhou, China; 2 Department of Breast Surgery, Aviation General Hospital, Beijing, China; 3 Department of Breast Medicine, The Affiliated Foshan Women and Children Hospital, Guangdong Medical University, Foshan, China; 4 Department of Cardiology, The Affiliated Guangdong Second Provincial General Hospital of Jinan University, Guangzhou, China

**Keywords:** breastfeeding duration, cancer epidemiology, dietary phytochemical index, dyslipidemia, mammary biology, nutritional status

## Abstract

**Background:**

Micronutrients, dietary phytochemicals, and lipid metabolism influence mammary gland growth, lactation efficiency, and breast cancer incidence. The present retrospective investigation evaluated these aspects using clinical, dietary, and biochemical data from women with established lactation and breast cancer outcomes.

**Methods:**

This retrospective analysis involved 800 women found through hospital electronic medical data. Dietary consumption of micronutrients and phytochemicals was evaluated via food frequency questionnaires and dietary recalls. Lipid metabolism was assessed via serum lipid parameters and fatty acid indicators. Outcomes involved mammary gland growth markers, lactation history and efficiency, mammographic density, breast cancer risk, and tumor features. Multivariable regression analysis corrected for demographic, reproductive, lifestyle, and clinical variables were applied.

**Results:**

Vitamin D deficiency was noted in 48.5% of subjects and was related with higher breast cancer incidence (adjusted OR = 1.68; 95% CI: 1.21–2.33). Breast cancer instances had decreased intakes of vitamin D, folate, selenium, zinc, and vitamin A compared with controls. Increased dietary phytochemical index (DPI) was linked with reduced breast cancer incidence (adjusted OR = 0.48; 95% CI: 0.32–0.71). Dyslipidemia was observed in 60.8% of individuals and was linked with elevated tumor grade and advanced stage. Extended periods of exclusive breastfeeding have been linked to a lower incidence of breast cancer.

**Conclusion:**

Micronutrient consumption, dietary phytochemical concentrations, lipid metabolism indicators, and lactation history show substantial relation with breast cancer probability, mammographic density, and tumor features in this population highlighting the importance of nutritional and metabolic markers in breast health outcomes.

## 1 Introduction

One of the most common tumors to be identified in women globally, breast cancer continues to be a significant cause of cancer-associated morbidity and mortality. According to epidemiological data, reproductive, metabolic, and dietary factors affect a woman’s chance of developing breast cancer throughout her life. Nutritional intake such as micronutrient consumption and dietary composition, has been evaluated in relation to breast tissue features, lactation outcomes, and breast cancer risk in observational investigations (1, 2). Micronutrients like zinc, vitamin D, vitamin A, folate, and selenium are necessary for healthy tissue maintenance, immunological response, and cellular metabolism. Micronutrient consumption and circulation concentrations have been found to vary between women with breast cancer and counterparts in observational studies (3). Numerous epidemiological studies have found inverse relationships between vitamin D status and breast cancer prevalence; however results vary by population. Vitamin D and the likelihood of breast cancer have been extensively researched (4, 5). Due to its function in DNA synthesis and repair, folate intake has also been assessed, and population-based studies have shown links between folate intake and the risk of breast cancer (6, 7). Zinc and selenium consumption have been the subject of prior research examining micronutrient shortages and breast cancer risk. These investigations have suggested that deficits in these trace elements may be associated with an increased risk of breast cancer, potentially because of their role in immunological response, cellular regulation, and antioxidant protection (8, 9).

Dietary phytochemicals, such as flavonoids and lignans present in fruits, vegetables, legumes, and whole grains, have been extensively studied in relation to the risk of breast cancer. Individuals who eat more foods high in phytochemicals are generally less likely to develop breast cancer, according to numerous cohort and case–control studies (10, 11). Dietary phytochemical indices are frequently employed as a realistic method to quantify total phytochemical consumption in population-based research to evaluate overall diet quality. When considered collectively, the data consistently shows a negative correlation between diets high in phytochemicals and the risk of breast cancer, underscoring the possible protective benefits of plant-based eating habits (12, 13).

The connection between lipid metabolism and features of breast cancer has been investigated in clinical and epidemiological research. Women with breast cancer have been found to have altered serum lipid profiles, including triglycerides, total cholesterol, low-density lipoprotein cholesterol, and high-density lipoprotein cholesterol (14, 15). Dyslipidemia may be more common in afflicted women and may be correlated with tumor grade and illness stage, according to retrospective data. Simultaneously, a number of studies have investigated the relationship between dietary fat intake—especially saturated fats and polyunsaturated fatty acids—and the risk of breast cancer; however, the findings are still inconsistent, highlighting the intricacy of lipid metabolism and its unclear influence on the onset of breast cancer (15, 16).

Mammographic density is regarded as a trustworthy intermediate indicator of breast cancer risk since it shows differences in the makeup of breast tissue. Mammographic density and nutritional variables, such as total dietary patterns and micronutrient intake, have been linked in observational research (17). Mammographic density has been specifically studied in relation to vitamin D status and other dietary factors; some research indicates that reduced mammographic density may be linked to increased intake of certain nutrients, including calcium, antioxidants, and vitamin D (18). Findings have differed throughout populations, though, suggesting that although nutrition may have an impact on the composition of breast tissue, the connection is still unclear (19).

Reproductive factors, especially the length and history of nursing, have a significant impact on the risk of breast cancer. According to epidemiological research, women who breastfeed are less likely to get breast cancer, and longer and exclusive nursing is linked to a higher risk decrease (20). Additionally, new research links diet and nutrient consumption to reproductive behaviors that may impact the risk of breast cancer by indicating that metabolic variables and nutritional status may impact lactation duration and efficiency (21, 22).

Even though micronutrient exposure, dietary phytochemicals, lipid metabolism, mammographic density, and breastfeeding history have each been assessed separately, fewer investigations have examined these variables together within a single group. Many studies have concentrated on individual exposures or outcomes, restricting evaluation of their combined relation with breast cancer incidence and related clinical features. Thus, the current retrospective investigation used integrated clinical, nutritional, and biochemical data to assess micronutrient consumption, dietary phytochemical intake, lipid metabolism parameters, breastfeeding efficiency, mammographic density, and breast cancer outcomes.

## 2 Methodology

### 2.1 Study design

This study was developed as a retrospective observational cohort study to investigate how micronutrients, phytochemicals, and lipid metabolism affect mammary gland development, lactation efficiency, and breast cancer risk. Due to the retrospective design, dietary exposures reflect anticipated habitual intake determined by previous documented data and therefore represent prior consumption patterns rather than prospectively evaluated dietary behavior. The researchers chose a retrospective study design because it enables them to investigate how long-term dietary patterns, biochemical markers, and clinical results correlate with data from medical records, nutritional databases, and cancer registries. This method follows traditional epidemiological approaches, which researchers use in nutritional oncology and maternal health studies.

### 2.2 Study population and sample size

The study included a total sample size of 800 women who were located through hospital electronic medical records and public health databases that were linked to the hospital system. Participants were selected based on the availability of complete nutritional, biochemical, lactation, and clinical outcome data. Women who had documented reproductive history and dietary intake records, lactation outcomes, and breast cancer diagnosis follow-up data made up the eligible participant group. Researchers established a fixed sample size of 800 for each retrospective analysis table, which helped maintain statistical consistency while enabling comparisons between different nutrient and metabolic domains.

### 2.3 Data sources and record retrieval

The researchers conducted retrospective data extractions from electronic health records, nutrition surveillance systems, laboratory databases, and cancer registry records at their institution. The researchers obtained dietary intake data from food frequency questionnaires (FFQs), 24-h dietary recalls, and dietitian-led nutritional assessments, which already existed. The FFQ utilized in this investigation was a semi-quantitative food frequency questionnaire that evaluated regular dietary intake via a preset list of frequently consumed foods, with standard portion sizes and frequency groups (e.g., daily, weekly, monthly), capturing habitual intake over 6 months. Standard food composition databases were used to translate dietary intake information from FFQs into daily mean nutrient intakes. Data for the 24-h dietary recalls came from clinical nutrition evaluations that were documented and included every food and drink that was ingested during the previous 24 h. When a subject had multiple 24-h recalls, the data were merged to account for daily (within-person) fluctuation and produce a more accurate estimate of typical dietary intake. The laboratory information systems provided access to all biochemical and lipid metabolism parameter information. The hospital oncology records and pathology reports established confirmation for breast cancer diagnosis and clinical staging.

### 2.4 Exposure assessment: micronutrients and phytochemicals

The study assessed micronutrient exposure through vitamins. The study examined phytochemical exposure through flavonoids, which people consume from fruits, vegetables, soy products, whole grains, and green tea.

Micronutrient intake levels (vitamin D, folate, vitamin A, selenium, and zinc) were quantitatively assessed from FFQ-based dietary information using recognized nutritional composition references. The dietary phytochemical index (DPI) was computed as the percentage of regular energy intake from foods high in phytochemicals and was classified into low, moderate, and high groups. The researchers established exposure categories through clinically relevant intake ranges that matched the intake patterns reported in previous epidemiological studies.

### 2.5 Lipid metabolism assessment

The researchers assessed lipid metabolism by analyzing serum lipid profiles and fatty acid biomarkers, which included total cholesterol, LDL-cholesterol, HDL-cholesterol, triglycerides, and omega-3 and omega-6 fatty acids and markers. These indicators functioned as surrogate markers to represent mammary gland lipid synthesis, lactation lipid composition, breast tissue physiology and carcinogenesis metabolic pathways.

Primary outcomes included indicators of mammary gland development (age at menarche and breast tissue density which exists) and lactation efficiency (duration of exclusive breastfeeding and milk volume indicators and reported breastfeeding success) and breast cancer outcomes (incidence and hormone receptor status and clinical stage at diagnosis). The researchers selected these outcomes because they showed biological plausibility and matched earlier results from retrospective studies and cohort studies that investigated maternal health and cancer epidemiology.

### 2.6 Covariates and confounding factors

The researchers obtained all potential confounding variables which included age, body mass index (BMI), parity, socioeconomic status, smoking history, physical activity, menopausal status and family history of breast cancer. The researchers included these covariates to reduce bias while establishing the actual effects of nutritional exposures on clinical outcomes. The adjusted analyses included age, BMI, and menopausal status because these factors may have an impact on lipid metabolism, nutritional intake patterns, and outcomes related to breast cancer. To adjust for differences in individuals’ reproductive and lifestyle factors, variables such as parity, smoking history, physical activity, and family history of breast cancer were also kept.

### 2.7 Statistical analysis

Descriptive statistics served to summarize demographic data and nutritional data and biochemical data and clinical data. The study presented continuous variables as means with standard deviations and categorical variables as frequencies with percentages. Multivariable regression models which suit retrospective data were used to evaluate the relationships between micronutrients and phytochemicals and lipid metabolism markers and outcomes. To assess relationships between nutritional exposures and clinical results, multivariable regression analyses were corrected for age, BMI, parity, smoking history, physical activity, menopausal status, and family history of breast cancer. Regression-driven models generated adjusted odds ratios with 95% confidence intervals. The study established statistical significance through standard thresholds which match the thresholds used in existing nutritional epidemiology research.

### 2.8 Ethical considerations

The study used de-identified patient records for its retrospective research according to institutional ethical standards. The study permitted bioethical waiver of informed consent because the analysis used existing clinical and nutritional data. The organization followed all data management practices according to standards for data protection and patient confidentiality.

## 3 Results

### 3.1 Baseline characteristics

The study population had an average age of 46.8 years with a standard deviation of 9.2 years. The cohort included 60.3% premenopausal women and 39.7% postmenopausal women. The average body mass index of the participants measured 26.4 kg/m^2^ with a standard deviation of 4.8 kg/m^2^. The study found that 24.5% of participants disclosed a family history of breast cancer. The reproductive characteristics of the study group showed that 76.5% of women had given birth and 68.0% had breastfed their children. The study found that 17.8% of women had used tobacco products at some point in their lives. The study found that 42.3% of participants showed minimal engagement in physical exercise. The baseline data show that the population consists mostly of middle-aged individuals who have undergone reproductive health treatment while displaying modifiable risk factors associated with their lifestyles (Table 1).

**Table 1:** Baseline characteristics of the retrospective cohort.

Parameter	Value
Age (years), mean ± SD	46.8 ± 9.2
Premenopausal women, *n* (%)	482 (60.3)
Postmenopausal women, *n* (%)	318 (39.7)
BMI (kg/m^2^), mean ± SD	26.4 ± 4.8
Family history of breast cancer, *n* (%)	196 (24.5)
Parity, *n* (%)	612 (76.5)
Breastfeeding history, *n* (%)	544 (68.0)
Smoking status, *n* (%)	142 (17.8)
Low physical activity level, *n* (%)	338 (42.3)

### 3.2 Micronutrient intake distribution in the study population

The study found that participants consumed 420 IU of vitamin D with a standard deviation of 180 IU, which resulted in 48.5% of the participants having vitamin D deficiency because they fell below the recommended dietary allowance (RDA) established for this vitamin. The participants showed sufficient folate intake because they consumed an average of 382 μg per day with a standard deviation of 110 μg, which resulted in 96% of the participants meeting the RDA requirements for this vitamin. The average daily vitamin A consumption reached 710 μg with a standard deviation of 260 μg, which resulted in 33.5% of women classified as deficient. The average selenium intake reached 48 μg with a standard deviation of 15 μg, which resulted in 22% of participants experiencing deficiency according to the RDA guidelines. The participants consumed 8.9 mg of zinc per day with a standard deviation of 2.4 mg, which resulted in 29% of participants not meeting the required intake. The study found that vitamin D deficiency affected most participants, while the study showed that most participants met folate requirements (Table 2).

**Table 2:** Micronutrient intake distribution in the study population.

Micronutrient	Mean intake ± SD	% RDA achieved	Deficient *n* (%)	Adequate *n* (%)
Vitamin D (IU/day)	420 ± 180	62%	388 (48.5)	412 (51.5)
Folate (μg/day)	382 ± 110	96%	214 (26.8)	586 (73.2)
Vitamin A (μg/day)	710 ± 260	79%	268 (33.5)	532 (66.5)
Selenium (μg/day)	48 ± 15	87%	176 (22.0)	624 (78.0)
Zinc (mg/day)	8.9 ± 2.4	81%	232 (29.0)	568 (71.0)

### 3.3 Dietary phytochemical index categories and energy intake

The cohort showed distribution of their Dietary Phytochemical Index (DPI) scores, with approximately 30.8% falling into the low DPI category, while 39.8 and 29.5%, respectively, achieved moderate and high DPI scores. The research found that daily energy intake decreased as participants moved from lower to higher DPI categories, which resulted in the low DPI group consuming 2,480 ± 410 kcal per day and the high DPI group consuming 2,145 ± 360 kcal per day. People consumed an average of 2.1 ± 0.9 and 2.8 ± 1.1 servings of fruits and vegetables each day. The results show that people who achieved higher DPI scores consumed less total energy while eating more plant-based foods, which led to a diet that contained more phytochemical-rich foods (Table 3).

**Table 3:** Dietary phytochemical index (DPI) categories.

DPI category	Score range	*n* (%)	Mean energy intake (kcal/day)
Low	<30	246 (30.8)	2,480 ± 410
Moderate	30–45	318 (39.8)	2,310 ± 390
High	>45	236 (29.5)	2,145 ± 360

### 3.4 Lipid profile characteristics of the study population

The study found that participants had an average total cholesterol level of 198 mg/dL with a standard deviation of 36 mg/dL, and their average LDL-cholesterol level reached 124 mg/dL with a standard deviation of 30 mg/dL. The average HDL-cholesterol level in the cohort reached 49 mg/dL with a standard deviation of 11 mg/dL, which showed that the participants possessed moderate levels of cardioprotective lipids. The average triglyceride concentrations showed different results because they had a mean value of 148 mg/dL with a standard deviation of 64 mg/dL. The study found that the n-6 to n-3 polyunsaturated fatty acid (PUFA) ratio reached 9.1 with a standard deviation of 3.4 because participants consumed more n-6 fatty acids than other fats. Participants consumed 11.8 ± 2.9% of their total energy intake from saturated fat while they consumed 6.9 ± 2.2% from PUFA and 12.6 ± 3.1% from monounsaturated fatty acids (MUFA). The lipid profile indicates that the participants show borderline dyslipidemia together with an unbalanced fatty acid distribution, which affects their metabolic health and breast health (Table 4).

**Table 4:** Lipid profile characteristics of participants.

Parameter	Mean ± SD	Reference range
Total cholesterol (mg/dL)	198 ± 36	<200
LDL-cholesterol (mg/dL)	124 ± 30	<100
HDL-cholesterol (mg/dL)	49 ± 11	≥40 (men); ≥50 (women)
Triglycerides (mg/dL)	148 ± 64	<150
n-6/n-3 PUFA ratio	9.1 ± 3.4	1:1–4:1
Saturated fat (% energy)	11.8 ± 2.9	<10% total energy
PUFA (% energy)	6.9 ± 2.2	5–10% total energy
MUFA (% energy)	12.6 ± 3.1	10–20% total energy

### 3.5 Breastfeeding history and lactation efficiency indicators

The study found 68.0% of women had breastfed at some point in their lives, and their average breastfeeding duration reached 9.6 months with a standard deviation of 6.4 months. The study found that 31.0% of the participants practiced exclusive breastfeeding for a duration of 6 months or more. The study found that 20.3% of women reported experiencing lactation-related problems, while 23.0% of women breastfed their infants until they reached 3 months of age. The cohort reported that 17.0% of its members experienced milk shortage according to their own assessments. The study found that 72.1% of participants met the nutritional needs of lactation through their vitamin A consumption, while only 38.5% of participants met the requirements for docosahexaenoic acid (DHA) intake. The study results show that high breastfeeding rates existed, but women showed substandard performance in breastfeeding length, breast milk production, and DHA nutrient levels (Table 5).

**Table 5:** Breastfeeding history and lactation efficiency indicators.

Parameter	Value
Ever breastfed, *n* (%)	544 (68.0%)
Mean breastfeeding duration (months), *n* (%)	9.6 ± 6.4
Exclusive breastfeeding ≥6 months, *n* (%)	248 (31.0)
Lactation difficulty reported, *n* (%)	162 (20.3)
Vitamin A adequacy, *n* (%)	577 (72.1)
DHA intake adequacy, *n* (%)	308 (38.5)
Early weaning (<3 months), *n* (%)	184 (23.0)
Self-reported milk insufficiency, *n* (%)	136 (17.0)

### 3.6 Micronutrient intake by breast cancer status

Breast cancer patients showed a significant decrease in vitamin D micronutrient intake when researchers compared their intake to that of the control group (368 ± 152 vs. 446 ± 188 IU/day; *p* < 0.001). The cases had lower folate micronutrient intake than the controls, who had a control group mean intake of 398 ± 112 μg/day (341 ± 98 μg/day; *p* < 0.001). The average selenium micronutrient intake showed a statistically significant decrease among breast cancer patients when researchers compared with control group (44 ± 13 vs. 50 ± 16 μg/day; *p* = 0.002). The zinc micronutrient intake pattern showed similarity to other substances because the cases had lower zinc intake than the control group (8.1 ± 2.2 vs. 9.2 ± 2.5 mg/day; *p* = 0.004). The cases showed a significant decrease in vitamin A micronutrient intake, which reached a daily average of 652 ± 241 μg compared to the control group, who averaged 734 ± 268 μg/day (*p* = 0.03). The results show that breast cancer patients have lower micronutrient consumption, while their insufficient micronutrient levels appear to increase their breast cancer risk (Table 6).

**Table 6:** Micronutrient intake by breast cancer status (*n* = 800).

Micronutrient	BC Cases (*n* = 214)	Controls (*n* = 586)	*p*-value
Vitamin D (IU/day)	368 ± 152	446 ± 188	<0.001
Folate (μg/day)	341 ± 98	398 ± 112	<0.001
Selenium (μg/day)	44 ± 13	50 ± 16	0.002
Zinc (mg/day)	8.1 ± 2.2	9.2 ± 2.5	0.004
Vitamin A (μg/day)	652 ± 241	734 ± 268	0.03

### 3.7 Association between dietary phytochemical intake and breast cancer risk

The low-DPI participants formed the base comparison group, which showed 43.0% breast cancer occurrence. The breast cancer risk reduction for women with moderate DPI scores reached statistical significance when compared to the control group (adjusted OR = 0.71; 95% CI: 0.50–0.99). The high DPI group showed the strongest protective effect, which resulted in 22.4% of breast cancer cases and an adjusted odds ratio of 0.48 (95% CI: 0.32–0.71). It was noticed that higher intakes of specific phytochemical subclasses led to lower breast cancer risk independent of other factors because both flavonoids (OR = 0.62; 95% CI: 0.44–0.86) and lignans (OR = 0.58; 95% CI: 0.39–0.82) showed this effect. The results show that dietary phytochemical intake leads to breast cancer risk reduction through a direct inverse relationship, which operates at different intake levels after correcting for major confounding variables (Table 7).

**Table 7:** Association between dietary phytochemical intake and breast cancer risk.

DPI category	BC cases *n* (%)	Adjusted OR*	95% CI
Low	92 (43.0)	1.00	Reference
Moderate	74 (34.6)	0.71	0.50–0.99
High	48 (22.4)	0.48	0.32–0.71
Flavonoid intake	—	0.62	0.44–0.86
Lignan intake	—	0.58	0.39–0.82

*Adjusted OR = 1 → No association, Adjusted OR > 1 → Increased odds of the outcome, Adjusted OR < 1 → Decreased odds (protective effect).

### 3.8 Retrospective association of lipid metabolism markers with breast cancer characteristics

The study found that elevated total cholesterol and LDL cholesterol levels increased both tumor grade and advanced tumor stage, particularly for Stage III-IV tumors. The researchers found that patients with high-grade tumors presented lower HDL cholesterol levels, while patients with advanced stages showed similar results. The researchers discovered that elevated triglyceride concentrations directly correlated with aggressive tumor characteristics and metastatic cancer spread. The study found that 60.8% of study participants had dyslipidemia, which occurred more often in women who had Grade III tumors and advanced cancer. The study found that higher body mass index resulted in higher tumor grade and stage advancement. The researchers discovered that prior usage of statin medications protected patients from developing advanced disease, which included lower-grade tumors. The study found that postmenopausal women produced higher lipid levels and experienced more severe tumor stage progression (Table 8).

**Table 8:** Retrospective association of lipid metabolism markers with breast cancer characteristics.

Parameter	*n* (%) or Mean ± SD	Median (IQR)	Clinical reference range	Association with tumor grade	Association with tumor stage	*p*-value*
Total cholesterol (mg/dL)	212.6 ± 38.4	210 (186–238)	<200 desirable	Higher levels observed in Grade III	Increased levels in Stage III–IV	0.021
LDL-cholesterol (mg/dL)	138.9 ± 34.7	136 (112–162)	<130 optimal	Positively associated with high grade	Higher in the advanced stage	0.015
HDL-cholesterol (mg/dL)	46.8 ± 11.2	45 (39–54)	≥50 protective	Lower levels in high grade tumors	Reduced in Stage III–IV	0.033
Triglycerides (mg/dL)	172.4 ± 66.9	165 (128–208)	<150 normal	Elevated in aggressive tumors	Higher in metastatic disease	0.009
Dyslipidemia status	130 (60.8)	—	Clinical diagnosis	More frequent in Grade III	More frequent in late stage	0.018
BMI (kg/m^2^)	28.1 ± 4.9	27.6 (24.8–31.2)	18.5–24.9	Higher BMI with higher grade	Associated with Stage II–IV	0.027
Prior statin use	57 (26.6)	—	Medication history	Lower grade predominance	Less advanced stage	0.041
Menopausal status	123 (57.5)	—	Clinical record	Higher lipid levels observed	Higher stage frequency	0.019

*Statistically significant at *p* < 0.05.

### 3.9 Nutritional factors and mammographic density

People who had adequate vitamin D intake showed a 38.2% rate of high mammographic density, which proved to be statistically significant according to *p* = 0.002. It was found that people who had adequate folate and selenium intake showed lower high-density patterns, which resulted in statistical results of 44.6% versus 55.4% (*p* = 0.01) and 41.1% versus 58.9% (*p* = 0.03), respectively. The study participants who achieved a high Dietary Phytochemical Index (DPI) level showed a significant decrease in their high mammographic density measurement, which stood at 29.5% compared to 70.5% (*p* < 0.001). People who consumed high amounts of n-6 fatty acids showed a higher rate of high mammographic density, which reached 62.0%, while only 38.0% showed this condition (*p* < 0.001). The researchers discovered that people who maintained proper micronutrient levels while following plant-based diets experienced lower breast density, but those who consumed diets high in n-6 fatty acids showed increased breast density, which serves as a known intermediate marker for breast cancer risk (Table 9).

**Table 9:** Nutritional factors and mammographic density.

Variable	High mammographic density (%)	Low mammographic density (%)	*p*-value
Vitamin D adequate intake	38.2	61.8	0.002
Folate adequate intake	44.6	55.4	0.01
Selenium adequate intake	41.1	58.9	0.03
High DPI	29.5	70.5	<0.001
High n-6 intake	62.0	38.0	<0.001

### 3.10 Lactation history and breast cancer risk

Women who had ever breastfed showed a significantly reduced risk of breast cancer (adjusted OR = 0.72; 95% CI: 0.54–0.96; *p* = 0.02). The study found that breastfeeding duration of more than 12 months provided greater protection against breast cancer (OR = 0.61; 95% CI: 0.42–0.89; p = 0.01). Women who breastfed exclusively for 6 months or more showed the lowest breast cancer risk (OR = 0.58; 95% CI: 0.39–0.85; *p* = 0.005). Early weaning before 3 months (OR = 1.42; 95% CI: 1.02–1.98; *p* = 0.04) and self-reported lactation difficulties (OR = 1.36; 95% CI: 1.01–1.84; *p* = 0.04) both increased the risk of breast cancer. The study results demonstrate that breastfeeding shows two important aspects that affect breast cancer risk (Table 10).

**Table 10:** Lactation history and breast cancer risk.

Lactation variable	Adjusted OR*	95% CI	*p*-value
Ever breastfed	0.72	0.54–0.96	0.02
≥12 months breastfeeding	0.61	0.42–0.89	0.01
Exclusive ≥6 months	0.58	0.39–0.85	0.005
Early weaning	1.42	1.02–1.98	0.04
Lactation difficulty	1.36	1.01–1.84	0.04

### 3.11 Multivariable regression of micronutrients and breast cancer risk

The research found that vitamin D deficiency leads to increased breast cancer risk with an adjusted odds ratio of 1.68 and a 95% confidence interval from 1.21 to 2.33. Folate deficiency showed a significant connection with increased risk because its odds ratio reached 1.44 and the 95% confidence interval ranged from 1.02 to 2.03, while selenium deficiency demonstrated a similar pattern with its odds ratio of 1.39 and 95% confidence interval from 1.01 to 1.92. Zinc deficiency exhibited a slight upward tendency, which did not reach statistical significance because its odds ratio stood at 1.31, and 95% confidence interval extended from 0.97 to 1.77. The research found that study participants who had obesity with BMI values equal to or above 30 kg/m^2^ experienced higher breast cancer risk, which had an odds ratio of 1.56 and a 95% confidence interval from 1.12 to 2.18. The study results demonstrate that inadequate intake of essential micronutrients, together with elevated body weight, leads to increased breast cancer risk, which operates as two separate risk factors (Table 11).

**Table 11:** Multivariable regression of micronutrients intake and breast cancer risk.

Variable	Adjusted OR	95% CI
Vitamin D deficiency	1.68	1.21–2.33
Folate deficiency	1.44	1.02–2.03
Selenium deficiency	1.39	1.01–1.92
Zinc deficiency	1.31	0.97–1.77
BMI ≥ 30 kg/m^2^	1.56	1.12–2.18

### 3.12 Dietary fat composition and breast cancer risk

The study found that breast cancer risk increased with saturated fat intake, which exceeded 12% of total energy intake (OR = 1.47; 95% CI: 1.08–2.01) and with higher trans fatty acid intake, which exceeded 1.5% of energy (OR = 1.63; 95% CI: 1.14–2.34). The research discovered that elevated n-6 polyunsaturated fatty acid (PUFA) intake was linked to increased risk, which reached an odds ratio of 1.32 and a 95% confidence interval that extended from 1.01 to 1.73. The research established that high n-3 PUFA consumption leads to breast cancer risk reduction, which reaches an odds ratio of 0.66 and a 95% confidence interval extending from 0.48 to 0.90. The study found that monounsaturated fat intake (MUFA) showed a trend that did not reach statistical significance to decrease breast cancer risk (OR = 0.79; 95% CI: 0.58–1.06). The results indicate that breast cancer risk depends on dietary fat quality because unsaturated fats protect against cancer, while certain saturated and trans fats increase cancer risk in breast cancer patients (Table 12).

**Table 12:** Dietary fat intake and breast cancer risk.

Fat type	Intake level	OR	95% CI
Saturated fat	>12% energy	1.47	1.08–2.01
Trans fat	>1.5% energy	1.63	1.14–2.34
n-6 PUFA >8% energy	High	1.32	1.01–1.73
n-3 PUFA ≥1.2% energy	High	0.66	0.48–0.90
MUFA ≥15% energy	High	0.79	0.58–1.06

### 3.13 Combined dietary patterns, lactation efficiency, and breast cancer risk

Women who followed a Western dietary pattern experienced the highest breast cancer risk, which reached an OR of 1.62 and displayed the least lactation efficiency at 54.1%. The group that followed a mixed dietary pattern functioned as the reference group, which displayed moderate lactation efficiency at 67.8%, together with a baseline risk that reached an OR of 1.00. Diets that contained a high amount of plant-based foods led to a significant decrease in breast cancer risk, which reached an OR of 0.54, together with an increase in lactation efficiency, which reached 78.6%. The combination of a high Dietary Phytochemical Index (DPI) and high n-3 PUFA intake showed the strongest protective association, with the lowest breast cancer risk (OR = 0.48) and highest lactation efficiency (82.1%). People who consumed diets that contained sufficient micronutrients experienced both a decreased risk of health issues with an OR of 0.59 and an increase in lactation efficiency that reached 75.4%. Plant-based nutrient-rich dietary patterns provide evidence that they decrease breast cancer risk while they enhance lactation efficiency (Table 13).

**Table 13:** Combined dietary patterns, lactation efficiency, and cancer risk.

Dietary pattern	BC risk OR	Lactation efficiency (%)
Western	1.62	54.1
Mixed	1.00	67.8
Plant-rich	0.54	78.6
High DPI + n-3 PUFA	0.48	82.1
Micronutrient-adequate	0.59	75.4

### 3.14 Sensitivity analysis of premenopausal women

The research examined how dietary patterns, nutritional elements, and reproductive factors relate to breast cancer risk through a study conducted with 482 premenopausal women who participated in the research. High Dietary Phytochemical Index (DPI) was strongly protective (adjusted OR = 0.44; 95% CI: 0.28–0.69), as was adequate vitamin D intake (OR = 0.61; 95% CI: 0.42–0.90) and high n-3 PUFA consumption (OR = 0.58; 95% CI: 0.39–0.86). Obesity, which occurs when body mass index reaches 30 kilograms per square meter or more, leads to higher breast cancer risk (OR = 1.71; 95% CI: 1.12–2.61). Women who had never breastfed showed a higher risk of developing breast cancer (OR = 1.48; 95% CI: 1.02–2.14). The study results demonstrate that plant-based diets with high nutritional value and breastfeeding continue to provide protective benefits for premenopausal women, while obesity and lack of breastfeeding create major health risks for this group (Table 14).

**Table 14:** Sensitivity analysis of premenopausal women.

Variable	Adjusted OR	95% CI
High DPI	0.44	0.28–0.69
Vitamin D adequate	0.61	0.42–0.90
High n-3 PUFA intake	0.58	0.39–0.86
Obesity	1.71	1.12–2.61
No breastfeeding	1.48	1.02–2.14

## 4 Discussion

This retrospective investigation assessed the relationships of micronutrient intake dietary phytochemical intake, lipid metabolism, mammographic density, lactation history, and breast cancer outcomes. The results show consistent statistical relationships between nutritional and metabolic factors and both breast cancer occurrence and tumor features, and are in line with previous epidemiological evidence showing a protective effect of breastfeeding. However, because the study was observational and retrospective, these interactions should only be viewed as associations; no conclusions about causality can be made. Furthermore, the current study design does not allow for a conclusive establishment of the temporal sequence between exposure and result.

### 4.1 Micronutrients and breast cancer risk

Women with breast cancer were found to have lower micronutrients intake or to have poorer nutritional status when compared to controls. In line with results from population-based research conducted in various regions, vitamin D insufficiency was very common. Inverse correlations between circulating 25-hydroxyvitamin D concentrations and the incidence of breast cancer, as well as correlations with tumor grade and stage, have been documented by epidemiological research (23). Mechanistically, vitamin D has been shown to control cell division, proliferation, and apoptosis by activating the vitamin D receptor in breast tissue. Nevertheless, measures of circulation vitamin D metabolites, receptor expression, and downstream signaling activity were not included in this investigation. As a result, any biological explanation in this dataset is inferential and cannot be verified directly. The noticed connection may also represent confounding by lifestyle, adiposity, or sun exposure, known factors that affect vitamin D status.

Lower folate consumption among patients with breast cancer is consistent with observational research showing links between folate intake and the risk of breast cancer, especially in people with low baseline intake. DNA synthesis and methylation depend on folate, and changes in folate availability have been shown to affect genomic stability in laboratory models (24). However, homocysteine, circulating folate levels, and epigenetic indicators including DNA methylation patterns were not evaluated in this investigation. Because of this, the proposed connection between folate intake and mammary tissue carcinogenic processes cannot be directly investigated in this population and should be regarded cautiously.

Additionally, those with breast cancer consumed less zinc and selenium. Earlier observational investigations have found that breast cancer individuals and controls had different zinc and selenium levels. Zinc is involved in DNA repair and immunological function, and selenium has been shown to affect antioxidant defense systems (25, 26). However, the current study is unable to determine if these nutrients have functional effects on tumor biology in this cohort without biomarker-based evaluation of oxidative stress or immune function. When taken as a whole, these results corroborate the current association data but do not prove causation.

### 4.2 Dietary phytochemicals and breast cancer outcomes

In the current study, a decreased likelihood of breast cancer was linked with greater exposure to dietary phytochemicals. This result is in line with cohort and case–control studies that show negative correlations between the risk of breast cancer and dietary patterns high in phytochemicals Dietary phytochemical indices, which represent overall food quality rather than specific nutrients, have been used to measure combined intake of flavonoids, lignans, and other plant-derived chemicals. Mechanistic research has documented that several phytochemicals can regulate estrogen metabolism, oxidative stress, and inflammatory responses (27, 28). Nevertheless, the current study did not directly evaluate these pathways, and the DPI is a composite dietary measure rather than exposure to particular bioactive compounds. Therefore, rather than being the result of isolated effects of phytochemicals, the observed correlation may represent total food quality and associated healthy lifestyle practices. It is impossible to rule out residual confounding from unmeasured dietary or behavioral factors.

### 4.3 Lipid metabolism and tumor characteristics

Women with breast cancer were often found to have dyslipidemia, which was linked to higher tumor grades and more advanced stages. Studies looking at the lipid profiles of patients with breast cancer, both cross-sectional and retrospective, have found similar correlations. Women with breast cancer have been shown to have different concentrations of triglycerides, low-density lipoprotein cholesterol, high-density lipoprotein cholesterol, and total cholesterol when compared to counterparts (29). According to experimental data, lipid signaling pathways and cholesterol metabolites may affect estrogen receptor function and tumor growth. Although circulating lipid measures were employed as systemic metabolic indicators in the current investigation, no direct evaluation of lipid metabolism in breast tissue, lipid utilization in the tumor microenvironment, or lipid-derived signaling molecules was carried out. Therefore, rather than being a verified biological mechanism, the correlation between systemic dyslipidemia and tumor aggressiveness should be seen as an indirect one. Despite the fact that dietary fat intake has been inconsistently linked to breast cancer risk in the literature, current data indicate that circulating lipid profiles may exhibit higher relationships with tumor features than reported dietary fat intake (30). However, this observation might be impacted by shared metabolic variables such obesity and insulin resistance, as well as reverse causation, which occurs when the presence or growth of a tumor changes systemic metabolism (31).

### 4.4 Mammographic density and nutritional factors

In the current investigation, dietary and metabolic variables were linked to mammographic density, a recognized intermediate measure of breast cancer risk. Although results are still inconsistent, observational studies have found correlations between mammographic density and micronutrient intake, especially vitamin D and dietary fat intake (32). The current investigation did not use quantitative imaging measurements or longitudinal evaluation of density changes; instead, it relied on classified mammographic density data. Furthermore, important biological mediators like insulin-like growth factors, sex hormones, and inflammatory markers were not assessed. As a result, even while the discovered correlations are in line with earlier research, they do not directly provide light on the molecular processes that connect breast tissue composition and nutrition.

### 4.5 Breastfeeding, lactation, and breast cancer risk

Numerous epidemiological studies have shown a negative relationship between breastfeeding length and history and the risk of breast cancer (20). Consistent with pooled analyses and large cohort studies, a longer period and exclusive breastfeeding were linked to better outcomes. Increased mammary epithelial cell differentiation and decreased cumulative estrogen exposure are two suggested explanations. Nevertheless, measures of hormonal profiles, lactation-related endocrine markers (such as prolactin and oxytocin), and structural alterations in breast tissue were not included in this investigation. As a result, mechanical explanations are still theoretical and rely more on outside data than on firsthand observations from this group. Lactation performance has been proposed to be influenced by food intake and metabolic condition. Although associations between nutritional factors and lactation efficiency were found in this study, it is difficult to establish functional relationships between diet, lipid metabolism, and lactation physiology due to the lack of direct measurements of fatty acid profiles, breast milk composition, or metabolic regulators. Thus, these connections ought to be understood as observable correlations (20, 33).

### 4.6 Implications

This study adds to the increasing amount of epidemiological data showing links between metabolic health, dietary habits, and breast cancer outcomes. A more comprehensive understanding of the factors influencing breast health is offered by the integrated assessment of micronutrients, phytochemicals, lipid metabolism, and lactation variables. However, a major obstacle to identifying mechanistic pathways is the lack of direct biochemical, molecular, and tissue-specific observations. To better understand the biological mechanisms behind these relationships, future research should emphasize prospective cohort designs and include metabolomic, hormonal, and molecular profiling. Furthermore, exposure characterisation and causal inference would be strengthened by objective biomarkers of nutritional status and periodic dietary evaluations.

### 4.7 Strengths

The present investigation made use of integrated laboratory, clinical, and nutritional data, enabling a thorough evaluation of exposures and results. One important strength is the concurrent assessment of lipid metabolism, mammographic density, breastfeeding history, micronutrients, and dietary phytochemicals. The relationships were easier to discern when clinically significant tumor features were included. There was enough power in the sample size to assess several parameters in a single analytical model.

### 4.8 Limitations

The retrospective approach limits interpretation to associations rather than conclusive cause-and-effect correlations and precludes causal inference. Therefore, it is impossible to definitively determine the time sequence between food exposures and breast cancer outcomes. Dietary intake was measured using self-reported instruments, which are subject to recall bias and measurement error. Specifically, the adoption of food frequency questionnaires (FFQs) raises the risk of dietary exposure misclassification and recall bias. As has been well stated in nutritional epidemiology, these methods rely on participants’ recollection and impression of habitual intake, which may result in both random and systematic reporting errors.

Furthermore, the study cohort was drawn from a single clinical setting, which may restrict the findings’ external validity and generalizability to larger and more varied populations with various dietary, healthcare, and demographic traits. Even after multivariable adjustment, residual confounding remains. The dataset’s retrospective nature further restricts the granularity of dietary data, involving possible variations in portion size estimation, food preparation techniques, and unreported supplement use, all of which could affect the classification of nutrient consumption. Differences in completeness of dietary data across individuals a may additionally led to measurement bias.

Moreover, it is difficult to separate the independent effects of particular nutrients or bioactive substances since dietary nutrients and phytochemicals are taken in as part of complicated dietary patterns rather than in isolation. Therefore, rather than single-nutrient connections, the observed associations might be the result of combination or synergistic dietary effects.

## 5 Conclusion

This retrospective investigation highlights how lipid metabolism, breastfeeding patterns, phytochemical-rich foods, and micronutrient status all influence breast health outcomes. The results indicate that poor lipid profiles, minimal exposure to dietary phytochemicals, and insufficient consumption of essential vitamins and trace elements are all persistently linked to unfavorable mammary gland features and an elevated risk of breast cancer. Conversely, extended periods of exclusive nursing and excellent nutritional profiles seem to provide protective effects. All of these findings lend credence to the idea that throughout the reproductive lifespan, changeable dietary and metabolic factors affect the risk of breast cancer and the severity of tumors. Therefore, incorporating metabolic monitoring and nutritional evaluation into women’s health programs may help enhance long-term breast health management, risk stratification, and mitigation in clinical settings.

## Data Availability

The raw data supporting the conclusions of this article will be made available by the authors, without undue reservation.
