# Correction: Role of micronutrients, phytochemicals, and lipid metabolism in mammary gland development, lactation efficiency, and breast cancer risk

**DOI:** 10.3389/fnut.2026.1910643

**Published:** 2026-08-25

**Authors:** Jia Qi, Xiaoyuan Liang, Xiangzhi Li, Jing Zhu, Xueping Li, Yaoyi Kong, Kanghua Huang, Jun Lai, Kunpeng Hu, Xulong Fan

**Affiliations:** 1 Department of Breast and Thyroid Surgery, The Third Affiliated Hospital of Sun Yat-Sen University, Guangzhou, China; 2 Department of Breast Surgery, Aviation General Hospital, Beijing, China; 3 Department of Breast Medicine, The Affiliated Foshan Women and Children Hospital, Guangdong Medical University, Foshan, China; 4 Department of Cardiology, The Affiliated Guangdong Second Provincial General Hospital of Jinan University, Guangzhou, China

**Keywords:** breastfeeding duration, cancer epidemiology, dietary phytochemical index, dyslipidemia, mammary biology, nutritional status

There was a mistake in Table 8 as published. Authors have incorrectly calculated the patient counts and their corresponding proportions. The corrected Table 8 appears below.

**Table 8:** Retrospective association of lipid metabolism markers with breast cancer characteristics.

Parameter	*n* (%) or mean ±SD	Median (IQR)	Clinical reference range	Association with tumor grade	Association with tumor stage	*p*-Value^*^
Total cholesterol (mg/dL)	212.6 ± 38.4	210 (186–238)	< 200 desirable	Higher levels observed in Grade III	Increased levels in Stage III-IV	0.021
LDL-cholesterol (mg/dL)	138.9 ± 34.7	136 (112–162)	< 130 optimal	Positively associated with high grade	Higher in the advanced stage	0.015
HDL-cholesterol (mg/dL)	46.8 ± 11.2	45 (39–54)	≥50 protective	Lower levels in high grade tumors	Reduced in Stage III-IV	0.033
Triglycerides (mg/dL)	172.4 ± 66.9	165 (128–208)	< 150 normal	Elevated in aggressive tumors	Higher in metastatic disease	0.009
Dyslipidemia status	130 (60.8)	—	Clinical diagnosis	More frequent in Grade III	More frequent in late stage	0.018
BMI (kg/m^2^)	28.1 ± 4.9	27.6 (24.8–31.2)	18.5–24.9	Higher BMI with higher grade	Associated with Stage II-IV	0.027
Prior statin use	57 (26.6)	—	Medication history	Lower grade predominance	Less advanced stage	0.041
Menopausal status	123(57.5)	—	Clinical record	Higher lipid levels observed	Higher stage frequency	0.019

^*^Statistically significant at *p* < 0.05.

A correction has been made to the **Results** section, 3.8 Retrospective association of lipid metabolism markers with breast cancer characteristics: “The study found that estrogen receptor-positive tumors had elevated cholesterol levels, but no link to tumor stage was discovered. The research findings demonstrate that breast cancer characteristics show a strong connection to disrupted lipid metabolism patterns” was removed. The correct paragraph reads as, “The study found that elevated total cholesterol and LDL cholesterol levels increased both tumor grade and advanced tumor stage, particularly for Stage III-IV tumors. The researchers found that patients with high-grade tumors presented lower HDL cholesterol levels, while patients with advanced stages showed similar results. The researchers discovered that elevated triglyceride concentrations directly correlated with aggressive tumor characteristics and metastatic cancer spread. The study found that 60.8% of study participants had dyslipidemia, which occurred more often in women who had Grade III tumors and advanced cancer. The study found that higher body mass index resulted in higher tumor grade and stage advancement. The researchers discovered that prior usage of statin medications protected patients from developing advanced disease, which included lower-grade tumors. The study found that postmenopausal women produced higher lipid levels and experienced more severe tumor stage progression (Table 8).”

The original version of this article has been updated.

